# Severity of electrophysiological alterations correlates with severity of illness in the early phase of critical illness polyneuropathy

**DOI:** 10.1186/cc11135

**Published:** 2012-03-20

**Authors:** R Nemes, Z Fülep, B Fülesdi

**Affiliations:** 1 University of Debrecen, Hungary

## Introduction

We aimed to investigate the early characteristics of critical illness polyneuropathy in surgical patients in a 5-day follow-up setting.

## Methods

Twenty critically ill patients were enrolled showing signs of systemic inflammatory response, sepsis or multiorgan failure featuring APACHE II score ≥12 on admittance aged 26 to 86 years. Routine noninvasive nerve conduction study of bilateral median and ulnar nerves was performed on a two-channel portable Keypoint Medtronic apparatus. Nerve conduction studies were performed on five consecutive days starting within at most 2 days after admittance, then weekly follow-up was carried out. Electrophysiological findings were compared to age-matched control group parameters.

## Results

On first examination, within 2 days following admission 17 of 20 (85%) patients showed signs of axonal type sensory-motor polyneuropathy. Medians of compound muscle action potential (CMAP) and sensory nerve action potential (SNAP) amplitudes of all nerves showed a significant decrease compared to control values (*P *< 0.001). During the 5-day study period four patients showed improvement. Sensory nerve fibres were less severely affected than motor fibers. The consecutive measurements revealed negative correlation with the severity of peripheral interstitial oedema determined by circumference of the elbow. Changes in CMAP and SNAP amplitudes also showed a negative correlation with daily rated APACHE II and SAPS II severity scores, and thus with patients' general condition.

## Conclusion

Electrophysiological alterations appear early after the development of critical illness [1-4]. Early electrophysiological investigations are advisory although results should be evaluated cautiously, as it is hard to differentiate between definitive lesions and temporary disorder caused by bioenergetic failure [3,5,6] of the nerve which tend to improve with normalisation of patients' condition.

## References

[B1] Tennilä A Intensive Care Med 2000 26 1360 1363 10.1007/s001340000586 11089765

[B2] Khan J Neurology 2006 67 1421 1425 10.1212/01.wnl.0000239826.63523.8e 17060568

[B3] Latronico N Crit Care 2007 11 R11 10.1186/cc5671 17254336 PMC2151880

[B4] Mohammadi B J Neurol 2008 255 265 272 10.1007/s00415-008-0722-0 18283402

[B5] Latronico N Lancet Neurol 2011 10 931 941 10.1016/S1474-4422(11)70178-8 21939902

[B6] Bolton CF Crit Care Med 1996 24 1408 1416 10.1097/00003246-199608000-00022 8706499

